# The Recommended Deltoid Intramuscular Injection Sites in the Adult Population: A Cadaveric Pilot Study With an Orthopedic Perspective

**DOI:** 10.7759/cureus.66391

**Published:** 2024-08-07

**Authors:** Sundip Charmode, Simmi Mehra, Abhishek Kumar Mishra

**Affiliations:** 1 Anatomy, All India Institute of Medical Sciences Rajkot, Rajkot, IND; 2 Orthopedics and Traumatology, All India Institute of Medical Sciences Rajkot, Rajkot, IND

**Keywords:** the cadaveric study, clinical trail, peripheral nerve injury, periosteal injury, deltoid intramuscular injection

## Abstract

Background and aim

The deltoid is a common site for intramuscular injections, but guidelines for administration lack standardization. Global researchers propose various techniques, and recent study reports indicate a 1.5-15% incidence of nerve palsies due to injections. This pilot cadaveric study aimed to standardize the deltoid intramuscular injection sites in the Southeast Asian population.

Methods

This cadaveric study of a two-year duration was conducted in the Department of Anatomy as an intramural research project in collaboration with the Departments of Anatomy and Orthopedics. In the first year of study, which was the pilot phase of the project, the available six cadavers, i.e., 12 upper extremity specimens were dissected. Anthropometric measurements of deltoid muscle along with the distance of underlying neurovascular structures like the axillary nerve and posterior circumflex humeral artery were measured from neighboring bony landmarks. This article presents the observations of the six cadavers studied in the pilot phase and shall be followed up by another article after the project.

Results

In adults, in anatomical position, the mean distances of the axillary nerve and posterior circumflex humeral artery from the mid-acromial point are 8.19±0.616 and 8.66±0.968 cm, respectively. The deltoid thickness at 3, 5, and 7 cm from mid-acromial point was observed to be 1.079±0.13 cm (0.5-1.78 cm), 1.599±0.12 cm (1-2.96 cm), and 1.815±1.0 cm (1.2-2.5 cm), respectively. The acquired qualitative and quantitative data were tabulated, graphically represented, and statistically analyzed.

Conclusions

The deltoid intramuscular injection (IMI) must be given at or below the level of the midpoint of the deltoid muscle, but never in the upper half. We recommend a site, 4 fingerbreadths/9 cm below the mid-acromion point as the safest site to avoid injury to any underlying neurovascular structures.

## Introduction

Globally, the deltoid is the preferred IM site in clinical practice [1]. Many other IM sites have been considered over the deltoid based on the risk of injury to the underlying vessels and nerves. However, a paucity of uniform guidelines and faculty advisories persists for IM administration by healthcare professionals [2]. The incidence of intramuscular injection-related peripheral nerve injuries ranges from 1.5% to as high as 15% [3]. These observations were confirmed by another study conducted at Piramal Dadabhai (PD) Hinduja Hospital and Seth Gordhandas Sunderdas (GS) Medical College, Mumbai, in 2019 by Desai et al. on 354 patients where the incidence of IM-induced nerve injuries was observed as 82.5% [4]. More than 50% of deltoid intramuscular injections (IM) are given indiscriminately by untrained staff in unregistered settings in clinical practice, especially in Southeast Asian countries [3,5]. In East Asia and the West, extensive research is done namely by Cheung et al., Chen et al., Kim et al., and Nakajima et al. [1,6-8]. In India, only two research studies namely Patra et al. and Gurushantappa et al. exist on the anthropometry of axillary nerve [9,10]. In a recent clinical trial conducted by Surraj et al. in the South Indian population, two sites - first, 1 cm above the deltoid insertion, and second, midway between the middle of the arm and the deltoid insertion - were reported to be safe for deltoid intramuscular injection [11]. As per our theories and experiences, we contradict the above-mentioned sites, as we fear injury to the periosteum of the humerus at any site close to the deltoid insertion. In addition to this, a systematic review was conducted by Charmode et al. in 2022 that confirmed a few deficiencies in IM guidelines issued by regulatory bodies in India and across the world [12]. This cadaveric study is targeted to validate our review findings thereby aiming to propose a safe site for deltoid IM with the least chance of injury to the underlying neurovascular structures by conducting the anthropometric measurements of the deltoid muscle and determining the sites of various neurovascular structures underlying or related to the deltoid muscle.

This article was previously posted as a preprint in medRxiv on June 16, 2024 (https://www.medrxiv.org/content/10.1101/2024.06.14.24308929v1).

## Materials and methods

Study design and population

This cross-sectional, observational study was conducted in the Department of Anatomy, in collaboration with the Department of Orthopedics. The study duration was two years after approval from the Institutional Ethical Committee. The primary objective of this study was to conduct anthropometric measurements of deltoid muscle like thickness, length, and width. In addition to this, another objective was to measure the location of the axillary nerve, anterior circumflex humeral artery, posterior circumflex humeral artery, sub-acromial bursa, and sub-deltoid bursa from the mid-acromial point. The study aimed to identify and recommend a safe site for deltoid intramuscular injection.

This article presents the observations of the six cadavers dissected within the first year of the study (also called as pilot phase) and shall be followed up by another article at the termination of this project (with the comprehensive findings). The six cadavers comprised five males aged 55, 56, 63, 67, and 79 years and one female aged 61 years. All the cadavers were voluntarily donated to the department. The cause of death in all of them was reported to be natural with no medicolegal instance, physical injury, organ transplantation, malignancy, or any history of major/minor surgery involving any body part observed. None of the cadavers had any history of physical trauma or showed any evidence of involvement in any trauma, particularly in the region concerned.

Sample size calculation

For the sample size calculation, the prevalence rate of symptomatic axillary nerve compression was taken as 5%. This was taken from the reference study by Surraj et al. in 2022 conducted at the Department of Anatomy of All India Institute of Medical Sciences (AIIMS) Bibinagar [11]. The formula to calculate the sample size in medical studies from the prevalence rate was proposed by Pourhoseingholi et al. in 2012 [13].

Using the formula n = Z x PQ/L^2^, the sample size (n) was calculated as 72.6 and rounded up to 73. Thus, the calculated sample size was 73 cadavers. Here, the prevalence of the condition (P) was 5, Q (calculated as 1-P) was 95, precision (L) was taken as 5% with a 95% confidence interval, and the power of the study was considered as 80.0.

It was decided to commence the project with the cadavers available in the department, i.e., six cadavers/12 specimens. The study shall continue till its complete period of two years and the final observations shall be updated by submitting another short communication or commentary.

Eligibility criteria

Eligibility criteria (inclusion and exclusion) for the cadavers were framed by the investigators. The inclusion criteria for cadavers were (i) embalmed human bodies using standard embalming techniques; (ii) specimens not displaying (or without a history of) any congenital/acquired deformity, metabolic disorders or malignancy, or any other debilitating disease; (iii) specimens without a history of/not displaying any fracture of the humerus, scapula, or pectoral girdle; (iv) specimens without a history of any surgery performed on the humerus, scapula, or pectoral girdle; and (v) specimens do not display any scar, injury, or deep wound on the arm and shoulder joint region.

The exclusion criteria for cadavers were (i) unembalmed human bodies; (ii) specimens displaying (or with a history of) any congenital/acquired deformity, metabolic disorders or malignancy, or any other debilitating disease; (iii) specimens with history of/displaying any fracture of humerus, scapula, or pectoral girdle; (iv) specimens with history of any surgery performed on humerus, scapula, or pectoral girdle; (v) specimens displaying any scar, injury or deep wound on the arm and shoulder joint region.

Ethical considerations

This study was conducted as phase 2 of an intramural-funded research project in the Department of Anatomy in collaboration with the Department of Orthopedics of the institute. The project was submitted and presented to the Research Review Board (RRB) and after its approval, it was forwarded to the Institutional Ethical Committee of the institute. The IEC approval was received on September 13, 2023, with protocol ID F-IM/15/2023. The approval letter reference number is O.W.No./AIIMS.RKT/IEC/54/2023, dated September 13, 2023. The study participants were the donated cadavers whose consent was implied so separate written/oral consent was not required. The data collected were kept anonymous.

Clinical Trial Registry India (CTRI) registration

After receiving IEC approval, the project proposal was submitted to the Clinical Trial Registry of India (CTRI), and the registration was returned on September 27, 2023. The reference number is REF/2023/09/072948, and the link to the CTRI is https://www.ctri.nic.in/. However, the Clinical Trial Registry India (CTRI) registration could not be completed as cadaveric studies are not included in the CTRI registry.

Data collection

To collect the general information about the cadavers, a specially designed data collection form (in English) was formulated to collect demographic data, such as age, gender, religion, occupation, education, family contact details, postal address, cause of death, medical history, surgical history of concerned part, diagnosis, mode of treatment received (conservative or surgical) for the condition, unilateral or bilateral involvement, and duration of the illness. The medical records were referred to collect this information. In the event of the information unavailable in the medical case records, the family members were contacted to extract this information. In addition to this, the anthropometry findings of the concerned body structure in the cadavers were recorded. Informed consent proforma was not required in this study as all the study participants, i.e., the cadavers voluntarily donated to the institute.

Data collection procedure

**Figure 1:**
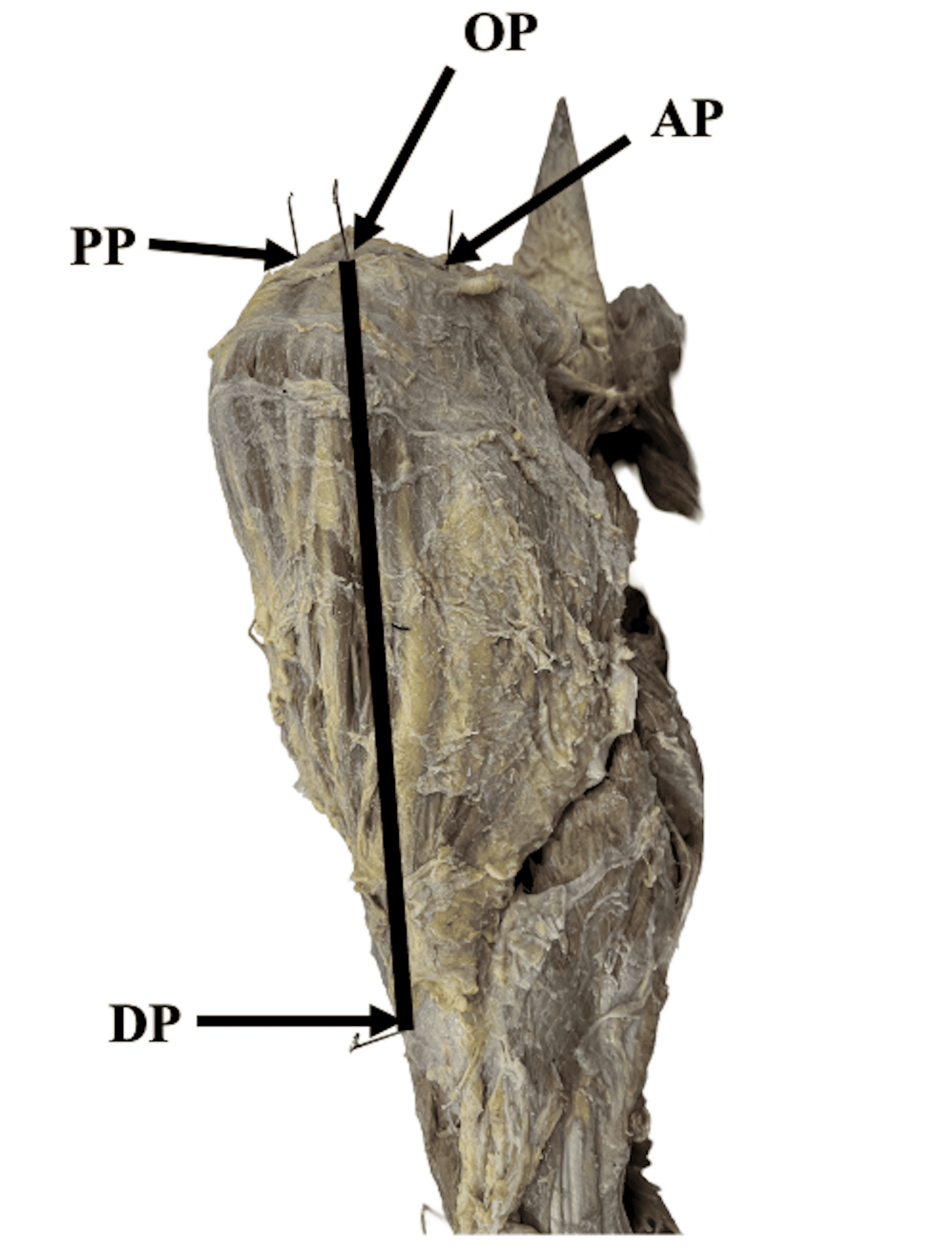
Bony landmarks for reference line measurements. The image is obtained from the Department of Anatomy, All India Institute of Medical Sciences (AIIMS) Rajkot, India.

Standard methods were used to dissect the concerned body region which included the cautious removal of skin along with subcutaneous tissue from the shoulder and arm region while retaining the fascia over the deltoid muscle. In this study, we focused on the acromial origin of the deltoid muscle (DM). The anthropometric measurements of the DM were done using digital vernier calipers and anthropometric tape. Each measurement was repeated with an accuracy of within 1 mm, and the average of both measurements, rounded to the nearest 1 mm, was accepted as the final result. Two investigators conducted the measurements separately on each cadaver at different times of the day. A research study piloted by Kim et al. on the Korean population was referred to as a reference study for the landmarks used for conducting anthropometric measurements in the present study [8]. Before taking the measurements, bony landmarks were identified and marked using demographic pens, and later after dissection with specimen marking pins on the concerned region namely the anterior point of the lateral border of the acromion (AP), the posterior point of the lateral border of the acromion (PP), and the acromion. The origin point (OP) was defined as the midpoint of a line connecting the AP and PP at the acromion level and a vertical line was constructed by connecting the OP to the lowest point of the deltoid tuberosity (DP). Figure 1 displays all these four landmarks as well as the length of the deltoid muscle. The thickness of the deltoid muscle, skin, and soft tissue was measured using digital calipers (resolution: 0.01/0.0005 mm, size: 1108-150 mm, range: 0-150 mm, accuracy: ±0.03 mm) at distances of 3, 5, and 7 cm from the OP. The length of the deltoid was measured from the OP to the DP. The distance of the anterior branch of the axillary nerve (AXN), and posterior circumflex humeral artery (PCHA) from the mid-acromial point were measured.

Data categorization

The data collected from the cadavers were categorized into reference line measurements (AP-PP, OP-DP), deltoid muscle thickness (at 3, 5, and 7 cm), and distance of AXN and PCHA from the mid-acromial point in anatomical and 60 degrees abducted positions of arm. The data were tabulated, graphically represented, and statistically analyzed.

Risk assessment

Two investigators measured every parameter individually and recorded their observations. Both digital vernier calipers and anthropometric tape or thread were used for the measurements and the difference was assessed. To tackle the interobserver bias, after completion of measurements of every specimen, both the investigators disclosed and discussed their findings and in case of more than 5% difference in their measurements, the third investigator was called upon to reconduct the measurements or to comment on the differences. To address the intraobserver variability, two readings of every variable were taken and then the average was calculated. To combat the environmental factors, measurements were taken in the mornings, afternoons, and evenings and again the average was calculated.

Data collecting instruments

Anthropometry instruments including digital Vernier calipers, anthropometric tape, dissection instruments, specimen marking pins, dermatographic pencils, plastic rulers, and stationery items were used for data collection.

Data analysis procedure

Data were analyzed using Microsoft Excel 2016 (Redmond, WA: Microsoft Corp.) and the mean and standard deviation were calculated. P-value was estimated. The t-test was used to compare the variables of interest according to sex, and a p-value less than 0.05 was considered statistically significant. The present study was conducted by the principles of the Declaration of Helsinki.

## Results

The project was divided into an initial pilot period followed by a continuation period. The objective of the pilot phase of eight months (from October 1, 2023, till May 30, 2023) was to dissect the available six cadavers, and the data arising out of these six cadavers, i.e., 12 specimens are presented in this study. The anthropometric data of the total 12 specimens were compiled in an Excel sheet, tabulated, graphically represented, and statistically analyzed as presented in Table 1. This shows that the length of the acromial border (AP-PP) and of the deltoid muscle (OP-DP) was higher in males compared to that in females.

**Table 1:** Reference line measurements (in cm). For the t-test, p-value <0.05 is considered significant. For the ANOVA test, p-value <0.01 is considered significant. SD: standard deviation; AP: the anterior point of the acromion; PP: the posterior point of the acromion; OP: the origin points of the deltoid muscle; DP: the lowest point of the deltoid insertion; S: significant; NS: not significant

Measurements	Total (mean±SD)	Males (mean±SD)	Females (mean±SD)	p-Value
Length of acromial border (AP-PP)	6.25±0.602	6.35±0.620	5.8±0.141	0.017 (S)
Length of deltoid muscle (OP-DP)	17.715±1.089	17.798±1.184	17.3±0.155	0.115 (NS)

As per Table 2, the thickness of the deltoid muscle was observed to be maximum (1.815 cm) at a distance of 7 cm from the origin point (OP) compared to distances of 5 cm and 3 cm. It was also observed that the thickness of 1.815 cm was maintained for a distance of 1 cm, between sites 7 cm and 8 cm from the OP.

**Table 2:** Measurements of the thickness of the deltoid muscle (in cm). SD: standard deviation

Measurements	3 cm, mean (range)	5 cm, mean (range)	7 cm, mean (range)
Deltoid muscle thickness	1.079 (0.5-1.78)	1.599 (1-2.96)	1.815 (1.2-2.5)

As per Table 3, the axillary nerve lies at a mean distance of 8.19 cm (7.5-10 cm) from OP in both males and females. The PCHA lies at a mean distance of 8.25 cm and 8.69 cm from OP in males and females, respectively. PCHA passes 0.5 cm below the axillary nerve on average.

**Table 3:** Location of the main branches of the axillary nerve and the posterior circumflex humeral artery in relation to the origin point (OP) (in cm). For the t-test, p-value <0.05 is considered significant. For the ANOVA test, p-value <0.01 is considered significant. SD: standard deviation; AP: the anterior point of the acromion; PP: the posterior point of the acromion; OP: the origin points of the deltoid muscle; DP: the lowest point of the deltoid insertion; S: significant; NS: not significant

Measurements	Total (mean±SD)	Males (mean±SD)	Females (mean±SD)	Minimum	Maximum	p-Value
Axillary nerve	8.19±0.616	8.19±0.681	8.19±0.0	7.5	10	0.115 (NS)
Posterior circumflex humeral artery	8.66±0.968	8.25±1.042	8.69±0.0	7.5	11	0.059 (NS)

As per Table 4, the axillary nerve lies at a mean distance of 7.09 cm (6.4-8.9 cm) from OP in both males and females. The PCHA lies at a mean distance of 7.59 cm and 7.58 cm from OP in males and females, respectively. AXN and PCHA moved 1.1 cm towards the acromion in the abducted position.

**Table 4:** Location of the main branches of the axillary nerve and the posterior circumflex humeral artery in relation to the origin point (OP) in 60-degree abduction (in cm). For the t-test, p-value <0.05 is considered significant. For the ANOVA test, p-value <0.01 is considered significant. SD: standard deviation; AP: the anterior point of the acromion; PP: the posterior point of the acromion; OP: the origin points of the deltoid muscle; DP: the lowest point of the deltoid insertion; S: significant; NS: not significant

Measurements	Total (mean±SD)	Males (mean±SD)	Females (mean±SD)	Minimum	Maximum	p-Value
Axillary nerve	7.09±0.616	7.09±0.681	7.09±0.0	6.4	8.9	0.0487 (NS)
Posterior circumflex humeral artery	7.56±0.968	7.59±1.042	7.58±0.0	6.4	9.9	0.059 (NS)

Table 5 enlists the various branching patterns of the axillary nerve observed (along with its relation to the posterior circumflex humeral artery) among the 12 specimens of the six cadavers dissected in the study. Type 1 and type 3 branching patterns were observed in four specimens each and found to be the most common in 33.3% of all the 12 specimens.

**Table 5:** Branching pattern of axillary nerve. AXN: axillary nerve; PCHA: posterior circumflex humeral artery

Type	Description	Number of specimens	Percentage of occurrence (%)
1	AXN passes above PCHA and gives 2 branches	04	33.3
2	AXN passes above PCHA and gives more than 2 branches	03	25
3	AXN passes below PCHA and divides into 2 branches	04	33.3
4	AXN crosses the PCHA from above to below	01	8.3

**Figure 2:**
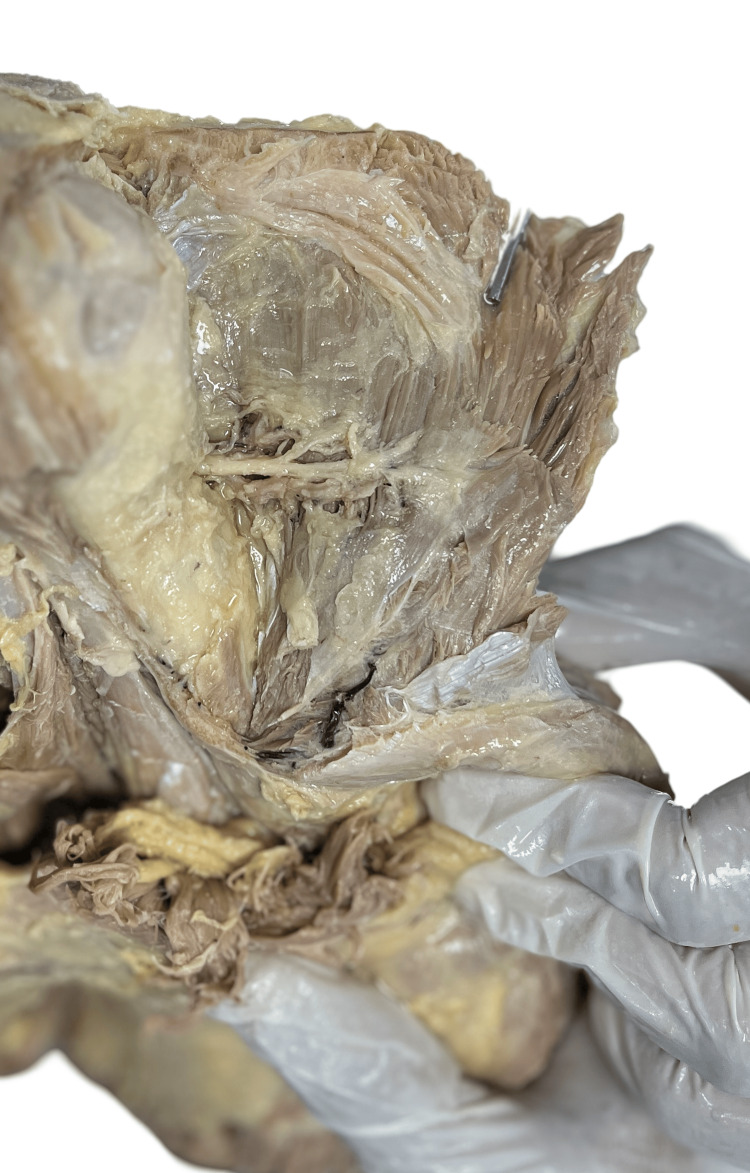
Specimen showing type 1 branching pattern of axillary nerve.

Figure 2 displays the type 1 branching pattern of the axillary nerve observed in specimens (right sides of cadaver numbers 1, 3, 4, and 5) in which the axillary nerve passes above the posterior circumflex humeral artery and gives two branches. This branching pattern of AXN is seen in four of the 12 specimens dissected, i.e., 33.3%. The distance between the AXN and PCHA remains the same throughout its course, from the quadrangular space till its termination.

**Figure 3:**
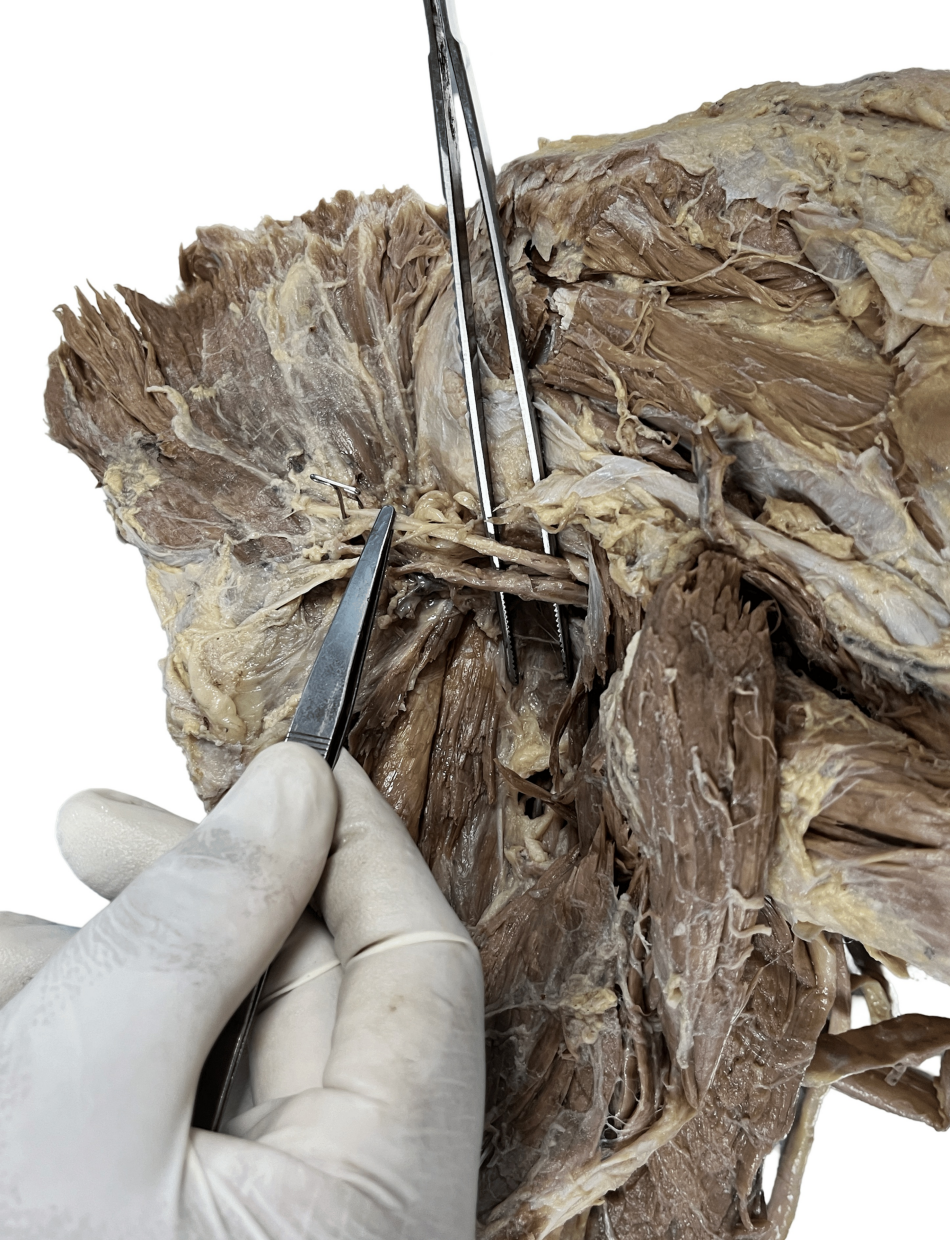
Specimen showing type 2 branching pattern of axillary nerve.

Figure 3 displays the type 2 branching pattern of the axillary nerve (in specimens 2, 6, and 9) in which the axillary nerve can be seen passing above the posterior circumflex humeral artery but ramifying more than twice. This branching pattern of AXN was seen in three of the 12 specimens dissected, i.e., 25%. The distance between the AXN and PCHA remains the same throughout its course, from the quadrangular space till its termination.

**Figure 4:**
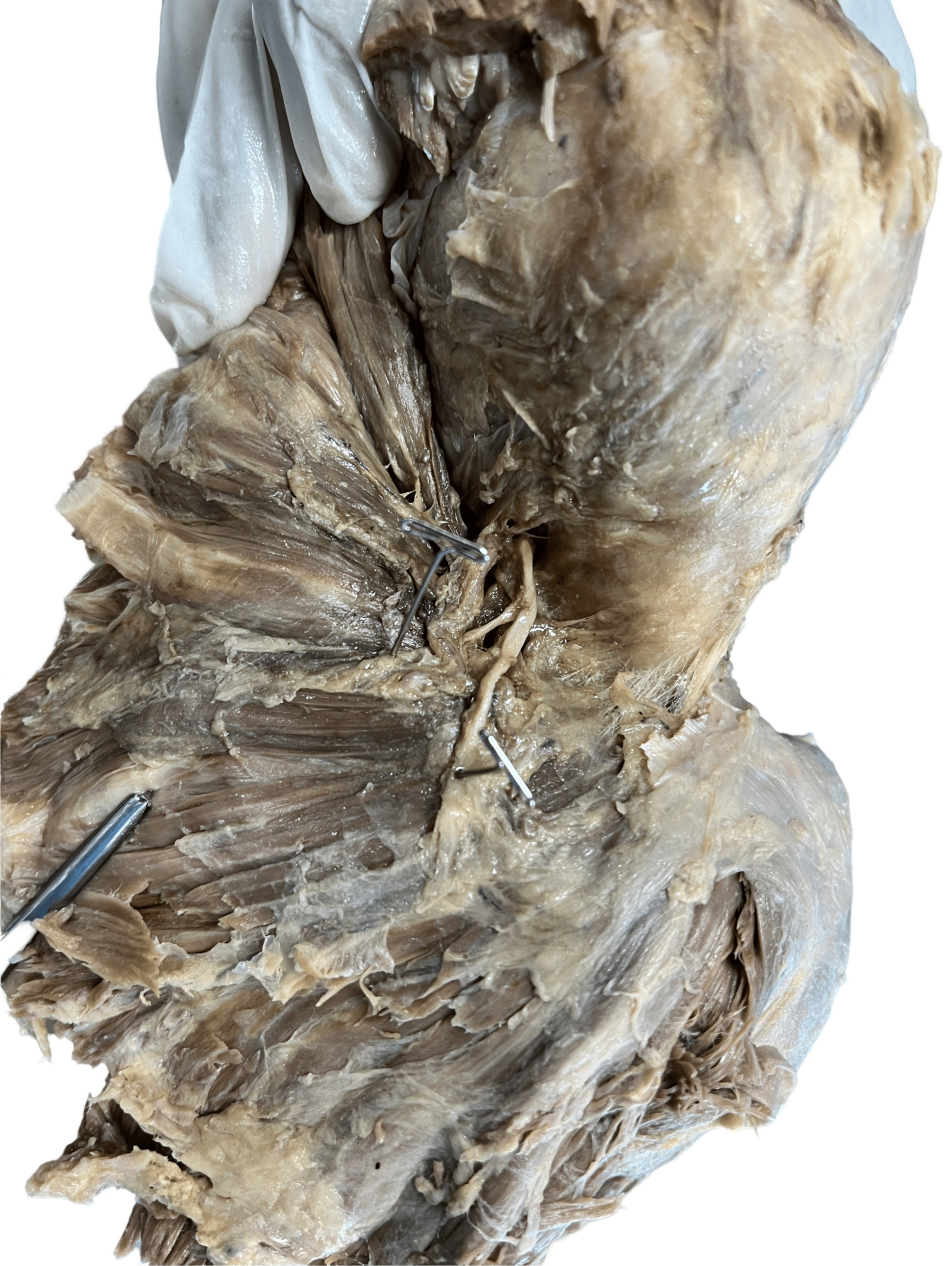
Specimen showing the type 3 branching pattern of axillary nerve.

Figure 4 displays the type 3 branching pattern of the axillary nerve (in specimens 7, 8, 10, and 11) in which the axillary nerve can be seen passing below the posterior circumflex humeral artery but ramifying into two branches. This branching pattern of AXN was seen in four of the 12 specimens dissected, i.e., 33.3%. The distance between the AXN and PCHA remains the same throughout its course, from the quadrangular space till its termination.

**Figure 5:**
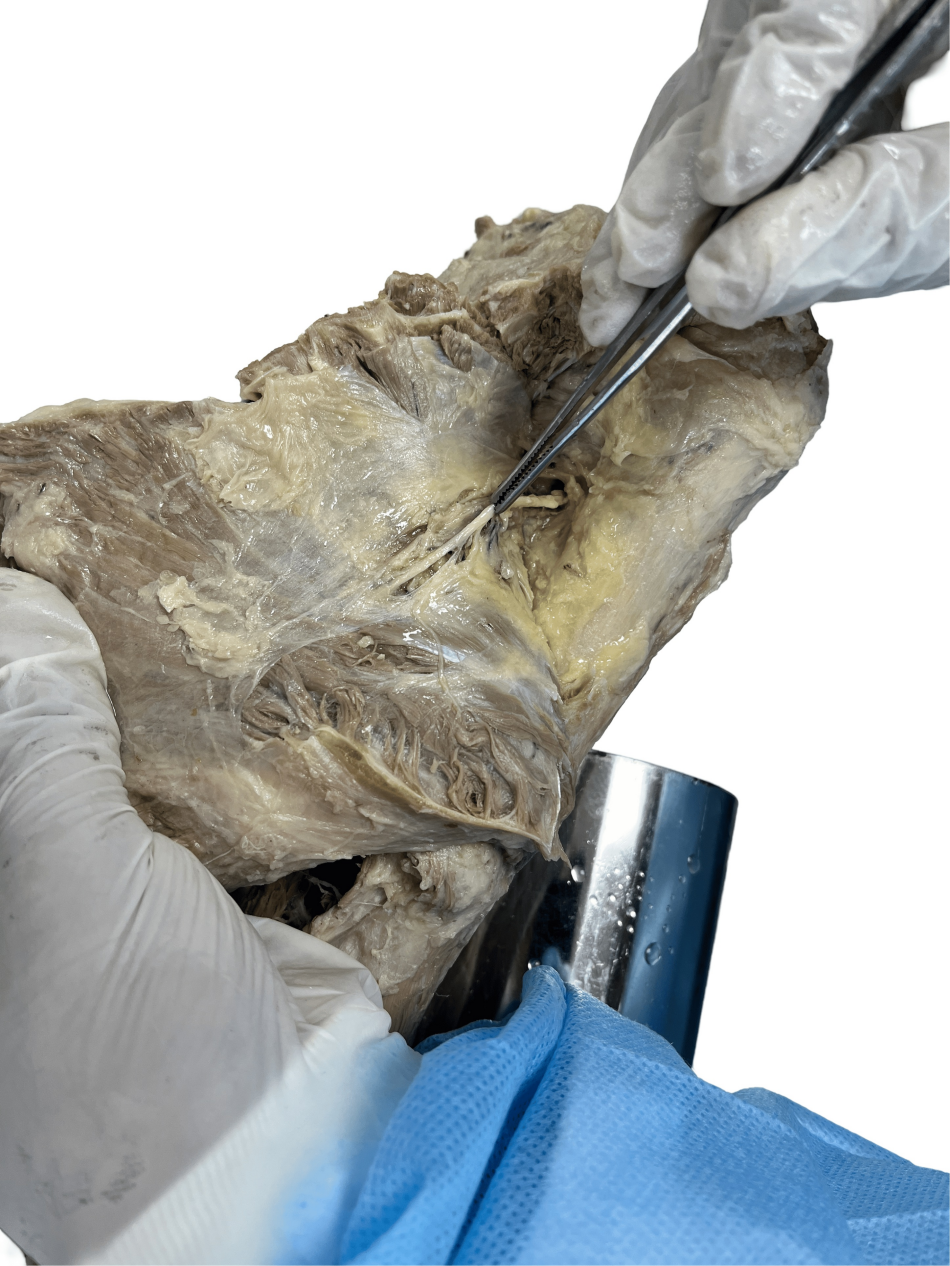
Specimen showing the type 4 branching pattern of axillary nerve.

Figure 5 displays specimen number 12 showing the type 4 branching pattern of the axillary nerve in which the axillary nerve can be seen passing above the posterior circumflex humeral artery and crossing it down. It is also seen ramifying into two branches. This branching pattern of AXN was seen only in one of the total 12 specimens dissected. The distance between the AXN and PCHA does not remain the same throughout its course, from the quadrangular space till its termination.

The linear regression coefficients are formulated using the observations from this study as follows: y = 1.132 x + 10.74 (where x is the length of the acromion {AP-PP} and y is the length of the deltoid {OP-DP}), and y = 0.0019 x + 8.16 (where x is the length of the deltoid {OP-DP} and y is the length of the acromion {AP-PP}). These equations allow us to determine the length of the deltoid muscle if the acromial length is known, and vice versa.

## Discussion

Kim et al. (Korea) and Nicholson et al. (USA) reported significant differences in the length of the acromion between males and females. Our results agree with those of these two studies [8,14]. Our findings do not match this study. Nakajima et al. (Japan) recommended the periphery of deltoid tuberosity as the safest point for deltoid IM [1]. We found this site extremely close to the periosteum, with the least thickness of the deltoid muscle. Gurushantappa and Kuppasad observed the distance of AXN from the midpoint of the posterolateral border of the acromion process to be 7.46±0.99 cm [10]. In the present study, it was 8.19 cm. Patra et al. (India) reported that the mean distance of AXN from the anterior and posterior border of the acromion was 5.22 cm and 4.17 cm, respectively [9]. The present study's mean length of AXN from OP was 8.19±0.616 cm. Compared with the study by Nakajima et al., PCHA's location differed from ours [1]. In the study by Nakajima et al. and our study, the PCHA was 6.8±1 cm and 8.66±0.9 cm from mid-acromion, respectively. Cheung et al. (USA) reported that the anterior branch of AXN moves 1.3-1.4 cm toward the acromion if the shoulder is abducted to 60 degrees [6]. In our study, AXN and PCHA moved 1.1 cm. Kim et al. (Korea) reported the mean distance of AXN and PCHA (0.5 cm below) from mid-acromial point to be 5.8±1.0 cm and 6.3±0.9 cm, respectively [8]. The mean length of the deltoid muscle was 16:1±1:0 cm and the deltoid muscle was thickest at 7 cm. In our study, the mean deltoid length was 17.7 cm, 1 cm longer. The deltoid muscle was thickest at 7 cm. AXN and PCHA were at 8.19±0.61 and 8.66±0.96 cm, respectively. Nakatani et al. (Japan) observed in their study that AXN is frequently positioned 5 cm below the mid-acromion point [15]. Our findings do not match this study.

International immunization advisory groups, namely The Centers for Disease Control and Prevention (CDC) (2021), and National Immunization Technical Advisory Groups (NITAGs) in Ireland (2020) and New Zealand (2020), recommend the central and thickest portion, approximately 2 inches (5.08 cm) below the mid-acromion, as the safest site for deltoid intramuscular (IM) injections [16]. As per our study findings, this site is the most dangerous. According to the Handbook of Safe Injection Practices, released by the National Centre for Disease Control (NCDC), Government of India, a site 1 to 2 inches (4.4 cm) below the acromion process is the safest site [17]. Our study observation contradicts this recommendation. United States Department of Health and Human Sciences Centres for Disease Control and Prevention (2017) reports that the safe site for deltoid IM is about 2-3 fingerbreadths below the acromion process and above the armpit level of the upper arm [18]. Our study observations contradict these recommendations, the reason behind this may be the racial differences in the deltoid muscle morphometry.

Limitations

The limited number of cadavers is the first limitation because of which we conducted a pilot study in the first half of the project. The movement of the axillary nerve and posterior circumflex humeral artery towards the mid-acromial point is best appreciated if we conduct a study involving a living person, instead of cadavers. Visualization of these said structures and their movement is possible to be seen while performing arthroscopic surgeries of the shoulder joint, in which the quadrangular space and its contents are encountered.

## Conclusions

Based on the anthropometric observations, it is imperative to administer deltoid intramuscular injections at or below the midpoint of the deltoid muscle to prevent potential injury to the axillary nerve and posterior circumflex humeral artery, which are located in the upper half of the muscle, approximately 8.19 cm and 8.66 cm from the origin point, respectively. The optimal site, where the deltoid muscle exhibits maximum thickness (1.815 cm), is situated between 7 and 8 cm from the origin point. We advocate for a designated injection site with 4 fingerbreadths, or 9 cm, inferior to the mid-acromion point to ensure safety. To further mitigate the risk of neurovascular damage, we recommend positioning the vaccine recipient with their hand on their hip, thereby abducting the shoulder to 60 degrees, facilitating an injection into the muscle's midpoint. This methodology harmonizes anatomical precision with clinical efficacy, ensuring the avoidance of critical neurovascular structures while optimizing intramuscular delivery.
